# Sensory Integration during Vibration of Postural Muscle Tendons When Pointing to a Memorized Target

**DOI:** 10.3389/fnhum.2016.00682

**Published:** 2017-01-13

**Authors:** Normand Teasdale, Mariusz P. Furmanek, Mathieu Germain Robitaille, Fabio Carlos Lucas de Oliveira, Martin Simoneau

**Affiliations:** ^1^Département de Kinésiologie, Faculté de Médecine, Université LavalQuebec City, QC, Canada; ^2^Centre de Recherche sur les Soins et les Services de Première Ligne de l’Université Laval (CERSSPL-UL)Quebec City, QC, Canada; ^3^Human Motor Behavior Laboratory, Department of Human Motor Behavior, The J. Kukuczka Academy of Physical EducationKatowice, Poland

**Keywords:** sensory integration, posture, vibration, vibration induced falling response, hand pointing

## Abstract

Vibrating ankle muscles in freely standing persons elicits a spatially oriented postural response. For instance, vibrating the Achilles tendons induces a backward displacement of the body while vibrating the tibialis anterior muscle tendons induces a forward displacement. These displacements have been called vibration induced falling (VIF) responses and they presumably are automatic. Because of the long delay between the onset of the vibration and the onset of the VIF (about 700 ms), and the widespread cortical activation following vibration, there is a possibility that the sensory signals available before the VIF can be used by the central nervous system to plan a hand pointing action. This study examined this suggestion. Ten healthy young participants stood on a force platform and initially were trained to point with and without vision to a target located in front of them. Then, they were exposed to conditions with vibration of the Achilles tendons or tibialis anterior muscle tendons and pointed at the target without vision. The vibration stopped between each trial. Trials with vision (without vibration) were given every five trials to maintain an accurate perception of the target’s spatial location. Ankle vibrations did not have an effect on the position of the center of foot pressure (COP) before the onset of the pointing actions. Furthermore, reaction and movement times of the pointing actions were unaffected by the vibration. The hypotheses were that if proprioceptive information evoked by ankle vibrations alters the planning of a pointing action, the amplitude of the movement should scale according to the muscle tendons that are vibrated. For Achilles tendon vibration, participants undershot the target indicating the planning of the pointing action was influenced by the vibration-evoked proprioceptive information (forward displacement of the body). When the tibialis anterior were vibrated (backward displacement of the body), however, shorter movements were also observed. Longer movements would have increased the backward response of the sensed body movement. Thus, it is possible that pointing actions were adjusted on the basis of the expected consequences of the planned pointing action to avoid a response that could have compromised postural stability.

## Introduction

Since the pioneering work of Goodwin et al. (1972), we know that vibrations to the tendon of a muscle produce sensations of limb movements. The vibration activates mainly the muscle spindle primary endings and gives rise to an instantaneous afferent pattern (Goodwin et al., 1972; Burke et al., 1976; Roll and Vedel, 1982). The primary endings respond to low amplitude vibrations in a one-to-one manner for vibration frequencies as low as 3 Hz up to 80 Hz. Cutaneous receptors also can contribute to the sensation (Collins and Prochazka, 1996; Aimonetti et al., 2007, 2012).

When a person stands freely with the eyes closed, vibrating the Achilles tendons generate sensory signals similar to those that would be observed if the body would be moving forward (Eklund, 1972; Gurfinkel et al., 1988; Roll et al., 1989a,b). The postural response following the onset of the vibration is in the opposite direction, towards the back as if it was generated to compensate the stretching signals of the calf muscles generated by the vibration of the Achilles tendons. A similar, but reversed response occurs when vibrating the tibialis anterior muscle tendons; the sensory signal indicates the body is moving backward and a forward postural response is observed. These postural responses have been described as “vibration induced falling” responses (VIF; Eklund, 1972, 1973). It is believed that VIF responses are involuntary and represent an automatic reaction originating from supraspinal centers (e.g., Eklund, 1972; Gurfinkel et al., 1988; Roll et al., 1989b). Several recent cerebral imaging studies have shown that a widespread cortical activity is observed when a muscle tendon is vibrated (for a review, Naito et al., 2016). The areas activated include the primary motor cortex, dorsal premotor cortex, supplementary motor area. Activation also occurs in somatosensory areas. The time course of VIF responses is quite slow. For instance, Quoniam et al. (1995) reported that the VIF response starts about 700 ms after the onset of the vibration on the tendon of the tibialis anterior. There are also reports of an initial faster (within 200 ms after vibration onset) but small center of foot pressure (COP) response in the direction opposite to that of the following (and related) main evoked postural displacement (Caudron et al., 2008). It has been suggested this initial response is automatic because it was observed when subjects self-triggered the vibration stimuli or when they could expect the onset of the stimuli through specific timing cues.

Because of the observed delay and the widespread cortical activation, there is a possibility that before the VIF response (which presumably is also automatic), an internal representation of the body (body scheme) is updated to indicate a change in the postural orientation. To examine this possibility, we asked subjects to point at a memorized target (hence without vision) from a standing posture. Specifically, if signals arising from vibrating the tendons reach higher level structures during the planning phase of the pointing movement, the motor commands for the upper arm should scale according to the muscles being vibrated when the movement is produced before the onset of the VIF. Hence, vibrating the Achilles tendons, because it gives rise to afferent signals indicating a forward displacement of the body, should lead to shorter movements than those without vibration. Alternately, vibrating the tibialis anterior tendons, because it gives rise to afferent signals indicating a backward displacement of the body, should lead to longer movements than those without vibration. Such results would provide strong support to the hypothesis that the sensory signals arising from the vibration induce an update of the internal representation of the body. This representation of the spatial position of the body could be available for the planning of motor commands of upper arm goal-directed movements.

## Materials and Methods

### Participants

Prior to commencing, participants were briefed about the experiment and provided a written informed consent. All forms and procedures were approved by the Human Research Ethics Committee of Université Laval. A total of 10 right-handed volunteers (6 males, 4 females; mean age: 24.3 ± 4.1 years old; mean body mass: 70.3 ± 11.9 kg; mean height 1.70 ± 0.10 m) with no known history of neurological or motor disorders participated in this investigation. After an initial analysis, data for one male participant were discarded because of systematic large overshooting of the target, even with vision of the target location.

### Task and Procedures

Participants stood barefoot on a force platform with their feet externally rotated by 10° and their heels 10 cm apart. Landmarks on the platform allowed maintaining a relatively constant position for all trials. In the start position, the participant’s right index finger touched the xiphoid process of the sternum with the elbow flexed and the arm at, approximately, 60° abduction. The contralateral arm was along the body. A target was located in front of the participant at about 80% of each participant’s arm length just below the height of the xiphoid process. For all trials, the task was to move rapidly the arm across the transverse plane and to stop with the right index finger just over the target with as much accuracy as possible. Participants were told to hold their index over the target for approximately 1 s before returning to the start position.

For each trial, a ready signal indicated the subjects to adopt the starting posture. The approximate initial anterior-posterior position and stability of the COP was verified visually on an oscilloscope (Hewlett-Packard 54601A) through monitoring the platform force moment around the mediolateral axis. For trials with vibration of the ankle muscle tendons, vibrators on both gastrocnemii or tibialis anterior muscles tendons were activated once the participant adopted a stable starting posture and the vibration lasted until the end of the trial. An auditory stimulus (4 kHz, 250 ms) presented 400 ms after the onset of the vibration was the imperative signal to point at the target. Assuming a mean reaction time of 300 ms, we postulated that most pointing actions would be planned before or around the onset of the VIF response. This is so because Quoniam et al. (1995) reported that the VIF response starts approximately 700 ms after the onset of vibration.

Each participant performed a total of 45 pointing movements with an interval of approximately 30 s between trials. All participants first pointed to the target without any vibration with full vision (Vision, five trials), and without vision (No Vision, five trials). Then, before each subsequent block of five trials, two trials with vision and without vibration were given to recalibrate the participants (i.e., to maintain an accurate perception of the target location). After another block without vision and without vibration, two blocks of five trials with vibration of the Achilles tendons and two blocks of five trials with vibration of the tibialis anterior muscle tendons followed. Trials with ankle vibration were performed without vision. Hereafter, the four experimental conditions are named Vision, No-vision, Vibration Achilles and Vibration TA.

### Instrumentation

Two custom-made vibrators served to stimulate the muscle proprioceptors. Each vibrator is made of a small DC motor inserted into a plastic cylinder (10 cm long, 3 cm in diameter). They generate a mechanical oscillation of 1-mm amplitude at a frequency of 80 Hz. The vibrators were securely fixed with rubber bands over the Achilles or tibialis anterior tendon.

A force platform (AMTI, OR-6 with MSA-6 MiniAmp, Watertown, MA, USA) fixed on the floor allowed recording the ground reaction force (Fz) and the moments around the sagittal (Mx) and frontal (My) axis. The platform was surrounded by a large wooden base (1.5 m wide × 2 m long) leveled with the platform. All signals were filtered (fourth-order zero-lag Butterworth filter, 7 Hz cut-off frequency) prior to calculating the COP.

An 8-camera video system (MaxPRO ver. 1.4.2.2, Innovision Systems Inc., Columbiaville, MI, USA) allowed recording the three-dimensional position of reflective markers placed bilaterally on the acromion, greater trochanter, lateral epicondyle of femur, lateral malleolus, and unilaterally (right side) on the lateral epicondyle of the elbow, styloid process of ulna and tip of the index finger. A target also was placed on a tripod in front of each participant just below the height of the xiphoid process at about 80% of each participant’s arm length. For the first two subjects, the markers were sampled at 100 Hz. Because the data processing increased the delay between trials, we then collected all subsequent data at 30 Hz.

Disposable self-adhesive surface electromyography (EMG) electrodes (Thought Technology Ltd., Uni-Gel^TM^ Single electrodes T3425, Montreal West, QC, Canada) were placed on the right side of the body on the anterior portion of the deltoid (AD), biceps brachi (BB), triceps brachii (TB), gluteus maximus (GM), semitendinosus (SEM) and on the left side for the tensor fasciae latae (TFL), biceps femoris (BF) and rectus femoris (RF). The ground electrode was placed at the olecranon process. The placements were defined in accordance the Surface EMG for Noninvasive Assessment of Muscles (SENIAM) guidelines (Hermens et al., 2000). All EMG signals were amplified at subject dependent gains (Octopus Bortec Biomedical Ltd., Calgary, AB, Canada) and analog band-pass filtered from 10 Hz to 1000 Hz.

A microcontroller (Basic Stamp^®^ BS2sx, Parallax Inc., Rocklin, CA, USA) served to control the timeline of each trial, the activation of the vibrators and the piezoelectric buzzer, and to synchronize recording of all data. All signals, but the kinematics, were recorded at 1 kHz with a 16-bit A/D converter (PCI-DAS6031, Measurement Computing, Norton, MA, USA).

### Data Analysis

**Figure 1:**
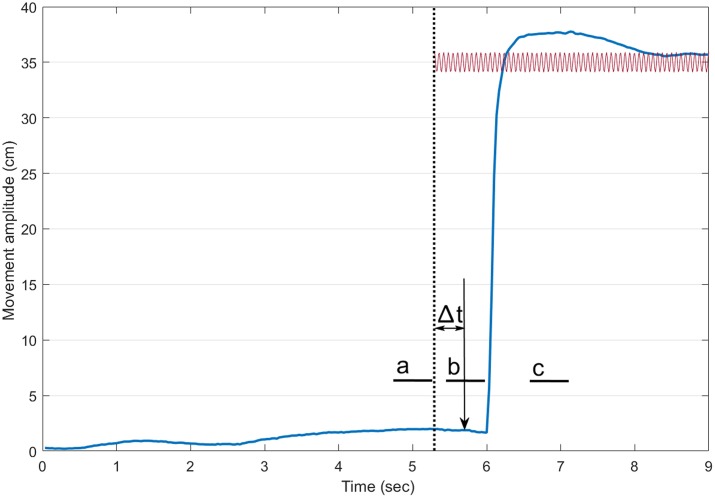
**Timeline of a trial with vibration.** The thick blue line illustrates the index finger displacement. The dotted vertical line shows the onset of the vibration. The sound (vertical arrow pointing down) arrived 400 ms after the onset of the vibration. The small thick horizontal lines indicate the three 500-ms periods where the mean center of foot pressure (COP) position along the antero-posterior axis was computed (a: before the onset of the vibration, b: before the onset of the pointing movement and c: at the end of the pointing). For trials without vibration, a similar timeline was adopted.

Data were analyzed using custom Matlab software (Matlab, the Mathworks Inc., Natick, MA, USA). Figure 1 presents the timeline for a trial with vibration. Reaction time was defined as the delay between the onset of the auditory stimulus and that of the index finger. The onset of the index was defined using a custom made algorithm detecting the first change in the signal above a baseline level (Teasdale et al., 1993). For each trial, the COP position of the participants along the antero-posterior axis was rebased using the mean position of the COP for a 500 ms period at the onset of a trial. To document the occurrence of the VIF response, the mean COP position then was computed for three different periods of 500 ms (a, b, c on Figure 1): (a) before the onset of the vibration (about 1.5 s before the onset of the movement for conditions without vibration); (b) just before the onset of the movement; and (c) when the participant’s index finger was stabilized at the endpoint. We hypothesized that the VIF response would be observed only during the third period (a backward position of the COP when the Achilles tendons were vibrated and a forward position when the tibialis tendons were vibrated).

The 3D positions of all markers were obtained using MaxPro (Innovision Systems Inc. Columbiaville, MI, USA). All kinematic signals were then imported in Matlab and synchronized with all other signals prior to further processing (fourth order low-pass zero-phase lag Butterworth filter with a cutoff frequency of 7 Hz). Constant error (CE) and absolute error (AE) were computed by comparing the end position of the index finger with that of the target. All statistical tests were performed with Statistica (version 12.0, StatSoft, Tulsa, OK, USA). Mean and standard error of the mean (SE) are presented throughout the manuscript.

## Results

### Reaction Time and Movement Time for the Pointing Movements

As stated above, for trials with vibration of the ankle tendons, an auditory stimulus to indicate participants to point at the target was given 400 ms after the onset of the vibration. With this procedure, we wanted participants to plan and initiate their pointing action before the VIF response. On average, reaction time was 419 ± 30 ms and it was unaffected by any of the vision or vibration conditions (*F*_(3,24)_ = 1.84, *p* = 0.165, *η*^2^ = 0.19). This indicates that on average, the delay between the onset of the vibration and that of the pointing action was 819 ms. Movement time for the pointing action also was not different across all conditions (on average, 353 ± 30 ms, *F*_(3,24)_ = 7.73, *p* = 0.067, *η*^2^ = 0.25).

### COP Along the Antero-Posterior Axis

**Figure 2:**
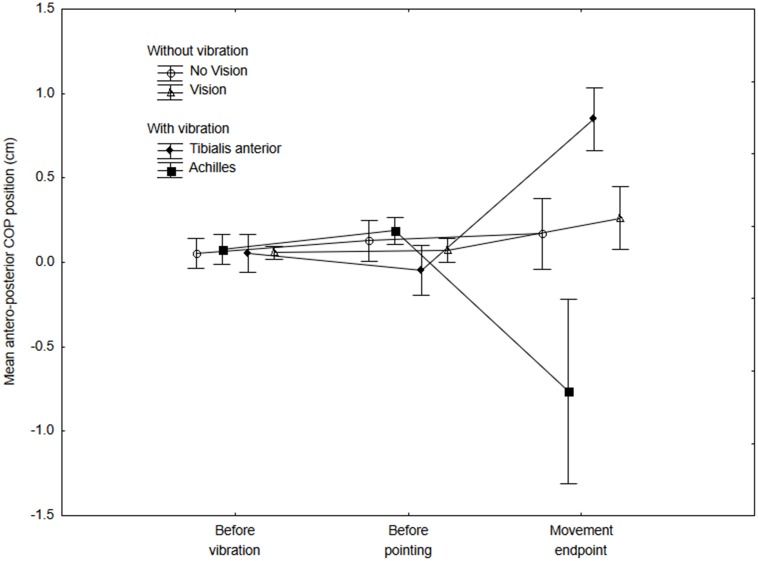
**Mean COP position along the antero-posterior axis at three different periods for the no-vibration (vision and no-vision) and vibration (Achilles and tibialis anterior) conditions.** The mean COP was calculated for the three 500-ms periods (i.e., before the vibration, before the onset of the pointing movement and at the end of the movement). Positive values indicate a forward COP position with reference to baseline values (that is, once a participant adopted the initial posture and showed a stable COP position within the first 4 s). Negative values indicate a backward COP position with reference to baseline values.

Figure 2 presents the mean COP position along the antero-posterior axis for the No-vibration (Vision and No-vision) and Vibration (Vibration Achilles, Vibration TA) conditions for the three periods analyzed (i.e., a, b and c on Figure 1). The ANOVA revealed a significant interaction of Condition × Period (*F*_(6,42)_ = 6.61, *p* = 0.000, *η*^2^ = 0.48). A difference between the conditions was observed for the last period only, that is, once the subjects had reached the target (i.e., after the end of the pointing action). As expected, the COP moved forward when the tibialis anterior tendons were vibrated while it moved backward when the Achilles tendons were vibrated (see Figure 2). It is important to note that, as expected, no difference in the COP position was observed before the onset of the pointing action. This suggests participants planned their movement before the VIF response had occurred.

### Amplitude and Accuracy of the Pointing Movements

**Figure 3:**
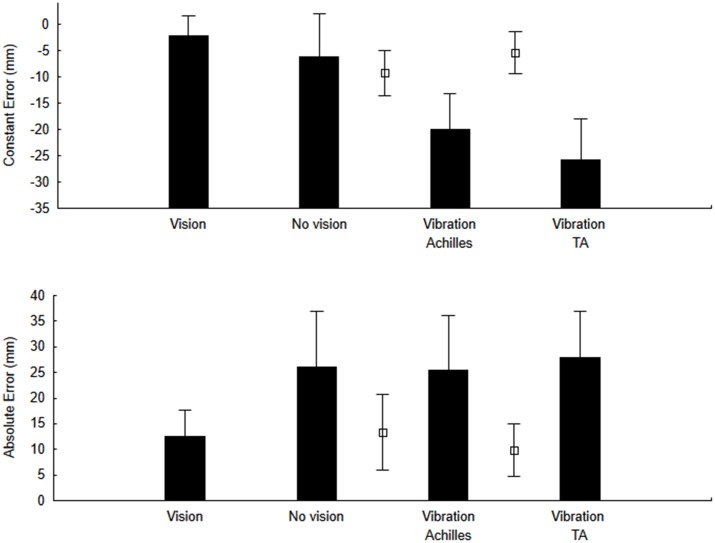
**Constant error (CE) and variable error (VE) for the no-vibration (vision and no-vision) and vibration (Achilles and tibialis anterior) conditions.** For comparison purposes, open square symbols depict mean values for the re-calibration trials (i.e., trials when pointing was performed with vision before each block of trials with vibration and without vision).

To test our main hypothesis, for the pointing actions, we calculated both the CE and the AE. The ANOVA for CE (Figures 3—upper panel) showed a significant effect of Condition (*F*_(3,27)_ = 3.79, *p* = 0.023, *η*^2^ = 0.32). As hypothesized, when the Achilles tendons were vibrated, the CE was negative (undershoot of the target). On the other hand, we did not observe a positive CE when the tibialis anterior tendons were vibrated as the CE was negative as well. For the AE (Figure 3—lower panel), the error was smaller with vision than for all other conditions but this difference was not significant (*F*_(3,27)_ = 2.27, *p* = 0.10, *η*^2^ = 0.22).

It could be argued that the negative CE observed when the Achilles tendons were vibrated resulted from a backward displacement of the body arising from the VIF response and not from a planned shorter hand movement. To verify this possibility, we computed the distance between the right acromion and the index finger. A shorter amplitude when the Achilles tendons were vibrated would indicate that the pointing response was shorter since the amplitude is now referenced to the body and not to an external referential. On average, the amplitude for the Vision, No-vision and re-calibration trials were 56.4, 55.6 and 55.8 cm, respectively. Mean values for these control conditions were compared using *T*-tests and all differences were non-significant (*t*_(8)_ = 1.03, *p* = 0.33 for the comparison between the Vision and No-vision conditions; *t*_(8)_ = −0.99, *p* = 0.35 for the comparison between the Vision and the re-calibration trials). The mean amplitude when the Achilles tendons were vibrated was significantly shorter than the mean amplitude of the preceding trials (on average, 53.97 cm when the Achilles were vibrated; *t*_(8)_ = 4.60, *p* = 0.001). This suggests that the negative CE (shorter amplitude) did not result from a backward movement of the body associated with the VIF but from a shorter pointing action with respect to the shoulder joint. The analyses for trials when the tibialis tendons were vibrated also showed a shorter pointing amplitude with respect to the shoulder joint (on average, 53.8 cm when the tibialis tendons were vibrated; *t*_(8)_ = 4.25, *p* = 0.002).

We also compared CE for the first pointing movement with vibration to subsequent trials. None of the participants had experimented the vibration before the first trial and a shorter amplitude for this trial could not be associated with a feedforward motor plan to avoid a potential destabilization. Specifically, we compared CE for the first trial with a vibration of the Achilles tendons (−3.4 ± 0.9 cm) to the mean of trials with (−0.4 ± 0.4 cm) and without vision (−0.6 ± 1.0 cm) preceding the first trial with vibration. Compared to trials with vision, the CE was negative (i.e., systematic undershoot of the target) and significantly smaller for the first trial with vibration of the Achilles tendons (*F*_(1,8)_ = 11.896, *p* = 0.008, *η*^2^ = 0.85). A similar result was observed when comparing the CE for the first trial with vibration of the Achilles tendons to those without vision (*F*_(1,8)_ = 7.29, *p* = 0.027, *η*^2^ = 0.65). These results suggest the vibration-evoked sensory information was integrated into the motor plan for the pointing action of the very first trial with vibration.

We conducted similar analyses for trials with vibration of the TA tendons, although participants had already experienced 10 trials with vibration of the Achilles tendons when they first were submitted to the first trial with the vibration of the TA tendons. The first trial with vibration of the TA tendons also showed a negative CE (−3.1 ± 1.1 cm), but the comparisons with trials without vision and with vision showed that these differences were not significant (*F*_(1,8)_ = 4.69, *p* = 0.064, *η*^2^ = 0.37, and *F*_(1,8)_ = 1.8, *p* = 0.216, *η*^2^ = 0.18, respectively for the comparisons with the Vision and No Vision conditions).

### EMG Activity

Finally, we examined the EMG activity for three muscles involved in the pointing action, the anterior deltoid, the biceps and TB. We specifically wanted to determine if the activity of the main agonist (TB) and antagonist (BB) muscles would show any modulation associated with the vibration of ankle tendons (for instance, smaller activity for the triceps and greater activity for the biceps when the Achilles tendons were vibrated). After a visual inspection of all data to insure EMG signals were normal within this period, data for one participant were removed because of irregular triceps signals. For the remaining eight participants, we calculated the $\int_{-50}^{100}EMG$ from 50 ms before the onset of the hand movement to 100 ms after the onset for each conditions. To allow between-subjects comparisons, the $\int_{-50}^{100}EMG$ obtained for the conditions No Vision, Vibration Achilles and Vibration TA were normalized using the same period from the condition Vision. Then, for each muscle, data for these three conditions were submitted to a one-way ANOVA. All ANOVAs were non-significant (*F*_(2,14)_ = 0.29, *p* = 0.75, *η*^2^ = 0.04, *F*_(2,14)_ = 2.46, *p* = 0.12, *η*^2^ = 0.26 and *F*_(2,14)_ = 1.75, *p* = 0.21, *η*^2^ = 0.20 for the anterior deltoid, BB and TB, respectively).

## Discussion

Vibration applied to ankle tendons is known to induce a body displacement in freely standing subjects and an illusory body tilt in restrained subjects. For instance, vibrating the Achilles tendon induces a backward body displacement in freely standing subjects and an illusory forward tilt when the body is restrained (Eklund, 1972; Kavounoudias et al., 2001; Adamcova and Hlavacka, 2007; Ceyte et al., 2007). The body displacement observed in freely standing subjects is presumed to represent an automatic reaction (e.g., Eklund, 1972; Gurfinkel et al., 1988; Roll et al., 1989b). In the present study, and as observed previously in freely standing subjects, vibrating the Achilles tendons led to a backward body displacement and conversely vibrating the TA tendons led to a forward body displacement. Importantly, in our study, whole body large displacements were observed after the pointing action and not before. No large VIF response was noted between the onset of the vibration and the onset of the pointing action. On average, the delay between these two events was 819 ms which is slightly longer than the latency of the body displacement after a tendon vibration (VIF response) reported by Quoniam et al. (1995). This first observation is important as it clearly suggests that the planning of the pointing action was made while vibration-evoked proprioceptive information could be interpreted by the central nervous system as a change in the postural orientation while this was not the case yet. The key question of this study was whether this information was included in the motor plan of the pointing action. Our hypothesis was that if this sensory information reaches higher level structures and is interpreted as a change in postural orientation during the planning phase of the pointing action, the amplitude of the movement should scale according to the muscles being vibrated. As expected, the amplitude of the pointing actions was shorter when Achilles tendons were vibrated. Vibrating the TA tendons, however, also yielded shorter movements while we expected longer movements.

There is a possibility that the shorter movements were not the result of integrating the postural orientation *per se* but rather a general response to conditions of postural instability. This has been proposed by Slijper and Latash (2004). In their study, they observed an increased co-contraction of distal muscles and reciprocal adjustments in trunk muscles when subjects were submitted to vibration of the Achilles tendons. They suggested these adjustments served to ensure equilibrium when subjects were submitted to conditions of postural instability (vibrating Achilles tendons being one of their conditions). In our study, movements of shorter amplitude and lower speed could have served to limit the instability induced by the pointing action. There are some arguments against this possibility, however. First, if the shorter amplitudes were a strategic response to control postural instability, slower movements could have been observed for the vibration conditions. This was not the case as movement times were similar for all conditions. Another argument is based on the lack of difference between the amplitude of the first trial with vibration of the Achilles tendons and the amplitude of pointing actions with and without vision. None of the participants had experimented the vibration before this first trial and a shorter amplitude for this first trial could not be associated with a feedforward motor plan to avoid potential destabilization.

The suggestion that sensory information from ankle proprioception and plantar sole mechanoreceptors serve to estimate body orientation when planning a motor response is in agreement with recent observations by Mouchnino et al. (2015). In this study, the authors measured the cortical response to somatosensory stimulation of cutaneous inputs during the planning phase of a step initiation and during its execution. They observed a facilitation of the P50-N80 SEP component in the early planning phase of the step initiation. The authors suggested that this mechanism could enhance the perception of cutaneous input leading to a more accurate planning and execution of the forces needed to unload the moving limb. In their study, the cutaneous inputs were directly involved in the motor action. Here, our results suggest that similar mechanisms may exist to update the motor plan of an upper-limb goal directed movement. For instance, the control of posture does not only serve the motor planning of an upper limb action by receiving feedforward commands and contributing through feedback processes once the movement is initiated. Rather, sensory information about body orientation during the actual planning of the upper arm movement can be integrated rapidly to modify and update the motor plan of the upper limb movement.

There is also a possibility that foot pressure distributions affected this result as it has been shown that vibration induced postural responses are dependent on the pressure distributions (Kavounoudias et al., 2001; Thompson et al., 2011). These authors proposed that ankle proprioception and plantar sole mechanoreceptors might be processed simultaneously following a vector addition mode to determine body orientation. For example, in Thompson et al. (2011) study, participants were submitted to a support platform perturbation (toes up or toes down) and the concurrent application of a vibration to the Achilles tendon and to the rear foot had differential effect on posture. Vibration of the Achilles tendon increased the influence of the rear foot vibration for toes up perturbations. On the other hand, it decreased the influence of the rear foot vibration when toes down perturbations were given. The authors suggested that this indicates the central nervous system uses both sources of sensory information to build a reference of verticality influencing the control of equilibrium during quiet and perturbed stance. A similar mechanism may have occurred in the present study and this could explain why we did not observe any overshoot when the TA muscle tendons were vibrated. In this particular case, the sensory signals from the TA (before the onset of the pointing action) suggested a backward movement of the body. A rapid pointing response of a greater amplitude would have produced a greater backward body movement that could have compromised postural stability. This was not the case when the Achilles tendons were vibrated as the sensory signals (before the onset of the movement) indicated a forward movement of the body allowing to produce a pointing movement of a shorter amplitude that did not compromise postural stability. Additional studies will be needed to examine if this could explain the shorter amplitudes observed when the TA tendons were vibrated (for example, by using movements of shorter amplitudes that would be less likely to destabilize subjects).

As for all studies, there are some limitations to this study. Presentation of the vibration conditions was not randomized. The main dependent variable in this study was the amplitude of the pointing action. The pointing action was made to a memorized target and included the usual motor variability associated with producing fast goal-directed motor actions (Schmidt et al., 1979; Harris and Wolpert, 1998; Scott, 2004). We felt that randomizing conditions could add some additional undesirable variability. This has been observed in several postural studies. For instance, Horak et al. (1989) showed that the scaling of a postural response (muscle and torque responses) to postural perturbation amplitudes disappeared when perturbation amplitudes were randomized. Martin et al. (2000) also reported that the uncertainty about a visual perturbation (double-step paradigm) modified the planning of pointing actions to a target.

In conclusion, our results suggest that the sensory signals evoked by vibrating Achilles tendons prior to the VIF responses are integrated into the motor plan of a pointing action. We did not observe longer movements when the tibialis anterior tendons were vibrated. This indicates subjects integrated both the sensory signals evoked by vibrating muscle tendons and the expected consequences of the planned pointing action.

## Author Contributions

NT and MS contributed to conception, design, preparation, analysis and interpretation of the data and writing. MPF contributed to conception, data collection and analysis and writing. FCLO contributed to data collection and analysis. MGR contributed to preparation and data analysis. All authors approved the final version.

## Funding

This research was supported by funding from the Natural Sciences and Engineering Research Council of Canada to MS and NT.

## Conflict of Interest Statement

The authors declare that the research was conducted in the absence of any commercial or financial relationships that could be construed as a potential conflict of interest.
